# Recent Advances in Immunotherapy for Patients with Head and Neck Cutaneous Squamous Cell Carcinoma

**DOI:** 10.3390/cancers14215325

**Published:** 2022-10-29

**Authors:** Adam Khorasanchi, Richard Wu, Kari Kendra, Claire Verschraegen

**Affiliations:** Division of Medical Oncology, The Ohio State University Comprehensive Cancer Center, Columbus, OH 43210, USA

**Keywords:** cutaneous squamous cell carcinoma, head and neck, immunotherapy, immune checkpoint inhibitors, Anti-PD-1, Anti-PD-L1, cemiplimab, pembrolizumab

## Abstract

**Simple Summary:**

Cutaneous squamous cell cancer is a form of skin cancer, which is typically found in older fair-skinned individuals with frequent sun exposure. Most patients present with limited disease and can be treated with a combination of surgery and/or radiation with favorable outcomes. A small percentage of patients present with more aggressive, widespread disease. Immunotherapy has dramatically improved outcomes and has become the preferred treatment option for these patients. In this review article, the rationale for using immunotherapy in patients with squamous cell skin cancer is discussed. A summary of the new treatment options currently being explored is also provided.

**Abstract:**

Cutaneous squamous cell carcinoma (CSCC) is the second most common non-melanoma skin cancer. A majority of patients present with localized disease, but some can present with locally advanced or metastatic disease. Most of these advanced cases occur in the anatomical head and neck region and are associated with more aggressive disease, necessitating prompt and effective treatment. Prior to the emergence of immunotherapy, systemic treatment options were limited to platinum-based chemotherapy and salvaged with targeted epidermal growth factor therapy. These therapies were associated with poor efficacy and increased toxicity in an often frail, older population. Immunotherapy has dramatically improved outcomes in this patient population due to its favorable side effect profile, durable treatment response, and improved overall outcomes. In this review, an overview of the recent advances of immunotherapy in the management of CSCC in the anatomical head and neck region is provided, with a focus on advanced presentations.

## 1. Introduction

Cutaneous squamous cell carcinoma (CSCC) is the second most common non-melanoma skin cancer, and most cases develop in the head and neck anatomical area (HNCSCC) [1,2]. The main risk factors of HNCSCC are chronic exposure to ultraviolet (UV) radiation, age, lighter skin complexion, and immunosuppression [3]. It is a disease of older age, more prominent in men than women, with an incidence of 450 cases per 100,000 person years in the US and a lifetime risk of about 10% [4]. Multiple primary cancers usually develop over time. In more than 90% of cases, HNCSCC is a localized disease, which can be successfully treated with curative surgery or radiotherapy (RT) [5], as described in the National Comprehensive Cancer Network (NCCN) guidelines [6,7]. Less than 5% of cases are classified as locally advanced unresectable or metastatic disease (mHNCSCC), for which systemic treatment is necessary [6]. Furthermore, CSCC within the head and neck region compared to other disease sites is associated with poorer outcomes. Specifically, tumors involving the ear, temples, and lips are associated with a higher risk of recurrent and metastatic disease [7]. Therefore, aggressive treatment of HNCSCC is necessary to achieve optimal outcomes. Other characteristics associated with a poor prognosis include large tumors (>2 cm diameter) with significant depth of invasion (>6 mm), perineural involvement, and poorly defined, undifferentiated tumors [7,8].

Until the advent of immunotherapy, systemic therapies for HNCSCC were limited to cytotoxic platinum-based chemotherapy and salvaged with targeted therapy inhibiting the epidermal growth factor receptor (EGFR) [9]. These therapies are associated with poor response rates, short duration of response, and substantial morbidity and toxicity, especially in patients of advanced age [10,11,12,13]. Since the Food and Drug Administration (FDA) approval of cemiplimab in 2018 and pembrolizumab in 2020, immunotherapy has become the standard of care for patients with locally advanced or mHNCSCC that is not amenable to curative surgery or RT [14]. These drugs have demonstrated overall response rates (ORRs) of approximately 40–50%, with higher ORRs seen in tumors with a higher mutational burden (TMB), due to increased neoantigen expression [14,15,16]. Interestingly, high TMB has been shown to predict response to immune checkpoint blockade in cancers (including lung, melanoma, and bladder) where there was a positive correlation between CD8 T cells and neoantigen load [17].

Immune checkpoint proteins serve as key regulators of the immune system’s response to cancer cells. T-cell activation requires a two-step process: (1) recognition of peptides via the T-cell receptor, and (2) interaction of coregulatory proteins (immune checkpoints) expressed on T cells, with partner proteins on tumor cells. Activation of these immune checkpoints may exhibit either stimulatory or inhibitory immune effects. Under normal circumstances, immune checkpoints ensure an appropriate immune response, while avoiding destruction of healthy tissue and immune hyperactivation as seen in autoimmune diseases. Programmed cell death protein-1 (PD-1) and cytotoxic T-lymphocyte-associated protein-4 (CTLA-4) are examples of immune checkpoints that have been studied extensively in cancer treatment. Upon activation, PD-1 and CTLA-4 deliver inhibitory signals to T cells resulting in T-cell inactivation. Immune checkpoint inhibitors (ICIs) block these proteins, resulting in an augmented anti-tumor immune response [18,19].

This review provides an overview of the recent advances of immunotherapy in the management of HNCSCC, with a focus on advanced presentations.

## 2. Methods

**Figure 1:**
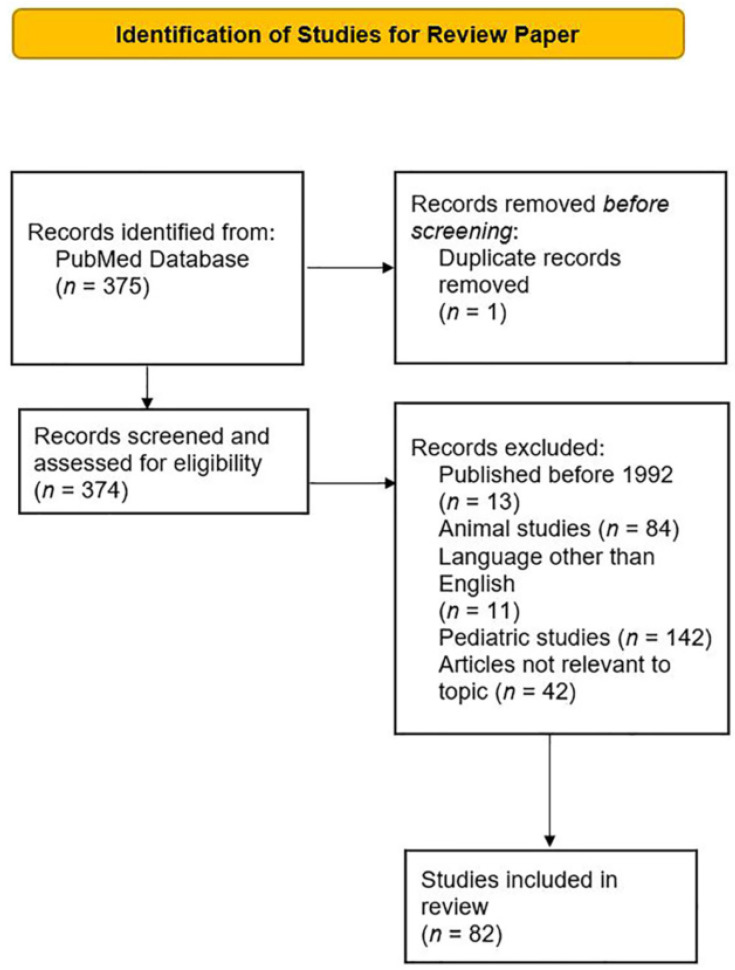
PRISMA flow diagram of literature search and study selection for review.

For this review paper, the search terms “immunotherapy” and “cutaneous squamous cell carcinoma” were used in the PubMed database. The search revealed 375 papers published between the years 1971 and 2022. These were screened for eligibility. One duplicate record was removed. Only articles written in English and published within the last 10 years were included. Articles that did not involve adult human subjects were excluded. Forty-two articles not relevant to this topic were excluded. A total of 82 articles were included in this review (Figure 1).

### 2.1. HNCSCC Carcinogenesis

Cutaneous squamous cell carcinoma arises from uncontrolled growth of atypical keratinocytes, a major cell type found in the epidermidis [20]. The current model for CSCC carcinogenesis is gradual and stepwise, in which chronic exposure to UV-B radiation/carcinogens may result in accumulation of tumor suppressor gene mutations. This can promote development of dysplastic precursor lesions, known as actinic keratoses (AK). However, a majority of AKs regress spontaneously, with regression rates of single AK lesions ranging from 15% to 63% after one year [21]. Only with the accumulation of subsequent gene mutations can AK further progress into squamous cell carcinoma in situ (SCCIS), then invasive CSCC [22]. The majority of invasive CSCC cases arise from progression of AK; however, they can also develop de novo [20].

UV-B radiation is a major risk factor for CSCC development and has several detrimental effects on skin cells. It is absorbed almost completely within the epidermal skin layer. Exposure to UV-B radiation is responsible for promoting skin inflammation, and excess exposure can result in keratinocyte cell injury and apoptosis [23]. UV-B radiation causes several types of DNA damage including point mutations (C→T and CC→TT), crosslinking (thymine and pyrimidine dimers), and strand breaks [23,24]. Consequently, DNA transcription and replication is impaired, leading to genomic instability and loss of function of tumor suppressor genes that normally promote senescence and apoptosis [23]. TP53 is the most common gene mutation in CSCC, followed by NOTCH1/2, FAT1, and CDKN2A [25]. This genomic instability indirectly promotes activation of downstream pathways such as PI3K/AKT/mTOR, which allow uncontrolled keratinocyte proliferation [20].

### 2.2. Role of Surgery in HNCSCC

Treatment for CSCC is guided by whether lesions are considered low or high risk. Lesions involving the head and neck are considered high risk regardless of their size. For high-risk CSCC, Mohs micrographic surgery (MMS) is the preferred treatment modality [7]. With MMS, a trained surgeon removes thin layers of skin followed by microscopic tissue visualization of each section. If cancer is visualized in a section, then this process is repeated until no further tumor is present. This process allows for complete removal of tumor prior to wound closure and is intended to preserve as much normal tissue as possible [26]. It is associated with high cure rates (99.3% local recurrence-free survival) as demonstrated in a 5-year prospective multicenter study involving patients with invasive CSCC [27]. Finally, MMS was found to be associated with lower recurrence rates (3%) compared to standard excision (8%) in a 2019 retrospective study by van Lee et al. [28].

If negative margins are achieved following MMS, but extensive perineural involvement or other high-risk features are present, adjuvant RT is suggested. However, for CSCC cases that lack these features, continued cancer surveillance is offered every 3-to-6 months for the first 2 years, then every 6-to-12 months for the next 3 years, followed by annual skin exams. If positive margins remain following MMS, re-excision should be attempted. It this is not possible, RT and/or systemic therapy with immunotherapy should be considered [7].

### 2.3. Role of Radiation Therapy in HNCSCC

Upfront RT is indicated for HNCSCC patients who are considered poor surgical candidates (inoperable or patient preference) or if surgery is infeasible. Surgery may not be appropriate for the following reasons: (1) potential disfigurement or loss of function, (2) impossibility to obtain clear margins, and (3) older patients with multiple comorbidities [29]. Definitive RT has been demonstrated to be effective in the treatment of invasive CSCC and is associated with low recurrence rates. In a 2012 meta-analysis of 14 retrospective studies (total of 1018 primary CSCCs), a 6.4% pooled average local recurrence rate was observed following RT [30].

Radiation therapy could be indicated to reduce the risk of recurrence following surgery. As previously discussed, adjuvant RT is indicated for patients with positive margins following surgery (lesions that cannot be re-excised); recurrence following prior surgery with negative margins; lymph node involvement, especially if an extranodal extension is present; or presence of disease with perineural invasion [7]. Adjuvant RT has been shown to improve survival outcomes in CSCC patients with positive margins [31,32,33,34]. Additionally, in CSCC cases with negative margins, adjuvant RT reduced the CSCC recurrence rate by 50% [35].

Radiation therapy is generally well tolerated. Potential RT-related toxicities are classified as acute (up to 6 months following RT) and delayed (greater than 6 months following RT). Acute toxicities manifest as skin reactions ranging in severity from redness to ulceration. Delayed toxicities can develop months to years following RT and are more frequently observed at higher dosages. Delayed toxicities may include changes in skin pigmentation, necrosis, atrophy, fibrosis, and secondary cancers [36].

Finally, the use of RT is contraindicated for genetic conditions such as Li-Fraumeni syndrome or ataxia-telangiectasia, which predispose patients to increased RT toxicity. Connective tissue disorders such as scleroderma are a relative contraindication due to the potential risk for RT-induced fibrosis and worsening of symptoms [37]. Given the high risk for complications, recurrent lesions should not be irradiated within the same radiation field [29].

### 2.4. Use of Chemotherapy in HNCSCC

There are no standardized chemotherapy regimens for the treatment of locally advanced or mHNCSCC. Chemotherapeutic agents that have been studied include platinum agents (cisplatin or carboplatin), 5-fluorouracil, bleomycin, doxorubicin, methotrexate, and taxanes. The most-utilized treatment strategies have been platinum agents, used as mono- or combination therapy. Chemotherapy efficacy data has been mostly limited to small observational studies with varying responses [38]. One randomized controlled trial involving treatment with a cisplatin-based regimen had an ORR of 34% [39]. Drawbacks for chemotherapy include: (1) poor efficacy, (2) a short duration of response (non-curative), and (3) increased toxicity, which is a major treatment barrier for older, frail patients with multiple comorbidities [40]. For these reasons, the NCCN guidelines recommend cytotoxic chemotherapy for patients who are deemed ineligible or experience disease progression following immunotherapy [7].

### 2.5. Use of Targeted Therapy in HNCSCC

EGFR inhibitors have been investigated extensively and represent a molecular target for the treatment of advanced HNCSCC. EGFR overexpression has been demonstrated in HNCSCC and may be associated with a poor prognosis [41]. EGFR inhibitors are classified into 2 groups based on their mechanism of action: monoclonal antibodies, which block EGFR, and small molecules that inhibit tyrosine kinase activity. Activation of EGFR within cells triggers downstream pro-proliferative pathways, including PI3K/AKT/mTOR and RAS/RAF/ERK. Cetuximab is a monoclonal antibody, which is the most studied of the EGFR inhibitors in the treatment of advanced HNCSCC. Its efficacy was first demonstrated in a phase II study, in which an ORR of 28% was observed at 6 weeks [39]. The NCCN guidelines recommend EGFR inhibitors for patients who are deemed ineligible for, or experience disease progression following, immunotherapy [7]. Panitumumab is another EGFR antibody, which demonstrated an ORR of 31% in a phase II study [42]. Finally, dacomitinib, a small-molecule pan-human EGFR, was evaluated in a 2018 clinical trial. Efficacy results were comparable to other EGFR inhibitors with an ORR of 28%, a median progression-free survival (PFS) of 6 months, and median overall survival (OS) of 11 months [43]. Unfortunately, EGFR inhibitors typically lack sustained antitumor responses as a result of acquired drug resistance, limiting their long-term efficacy. Drug resistance may develop for a variety of reasons, including overexpression of other cell surface receptors such as vascular endothelial growth factor receptor (VEGFR), mutations in the ATP-binding domain of the EGFR receptor, and mutations in downstream pathways [44].

An additional molecular target currently being explored in HNCSCC is the mitogen-activated protein kinase/ERK kinase (MEK) pathway, which is downstream from the EGFR pathway. A phase II study is investigating the combination of MEK inhibitor cobimetinib with atezolizumab in patients with advanced tumors, including CSCC. Results of this study (NCT03108131) have not been published yet.

Another molecular target is the VEGFR, which is believed to contribute to EGFR resistance. A phase I study investigating lenvatinib, a tyrosine kinase inhibitor of the VEGFR, in combination with cetuximab for advanced HNCSCC was recently completed (NCT03524326); the results have not been published.

Finally, the PI3K/AKT/mTOR downstream EGFR signaling pathway represents another promising therapeutic target for CSCC [45]. LY3023414 is a PI3K/AKT/mTOR inhibitor that has been shown to inhibit human CSCC cell growth in preclinical studies [46]. Additionally, GDC-0084, a small-molecule PI3K-mTOR inhibitor, has also been shown to inhibit human CSCC cell growth in preclinical testing [47].

### 2.6. Rationale for Immunotherapy in HNCSCC and Historical Perspective

The first immunotherapy drug used for the treatment of CSCC was intralesional interferon [48]. Over the past 3 decades, there has been increased knowledge regarding the immune system’s key role in CSCC pathogenesis [49]. The increased risk of HNCSCC in immunosuppressed patients suggests that natural immunosurveillance plays a major role in suppressing HNCSCC development [50].

Under normal circumstances, the immune system acts as a natural defense against cancer. The strength of immunosurveillance is primordial to prevent cancer growth. The healthy immune system operates in an elimination mode, by recognizing tumor-specific antigens, eliminating cancer cells, and preventing tumor growth. The immune system and tumor may enter an equilibrium mode, in which the cancer is allowed to persist without growing. An unhealthy immune system allows mutations to arise, and subsets of cancer cells then evade immune detection because of the dampened immune system’s antitumor response. This is known as the escape mode, which allows for uncontrolled cancer growth and progression [51,52]. Examples of unhealthy immune systems include patients with impaired T-cell function, who are unable to mount an antitumor response, and therefore are at increased risk of developing CSCC [53]. The overall risk for CSCC is up to 200-fold greater in solid organ transplant recipients (SOTR) compared to immunocompetent patients [54]. HIV patients are 2.6 times more likely to develop CSCC, and this likelihood increases with lower CD4 counts [55,56]. Patients with hematologic cancers such as chronic lymphocytic leukemia are 8-to-10 times more likely to develop CSCC [57,58].

Additionally, patients with advanced HNCSCC typically exhibit high TMB, a majority of which are caused by UV radiation exposure [59]. As previously discussed, UV radiation can cause P53 alterations, which in turn lead to defects in DNA repair. Collectively, these mutations can lead to the activation of downstream pathways including EGFR, MAPK, and PI3K/mTOR, which contribute to HNCSCC tumorigenesis. Epigenetic dysregulation via abnormal DNA methylation, histone modification, chromatin remodeling, and micro RNAs also promote HNCSCC carcinogenesis [60,61]. The high TMB exhibited in HNCSCC leads to higher levels of neoantigen formation and increases the likelihood of response to immune checkpoint inhibitor (ICI) therapy [9,14].

### 2.7. Approved Immunotherapy Agents for HNCSCC

Locally advanced HNCSCC is defined as a tumor that is no longer amenable to curative surgery or RT, or could lead to severe anatomical dysfunction when treated with surgery and/or RT [62]. Such aggressive local therapies can cause anatomical deformities and long-term psychosocial issues for patients. Metastatic HNCSCC is defined as stage III, IVA, or IVB on the tumor-node-metastasis (TNM) staging system [63].

In the following section, we describe the results obtained in well-designed clinical trials of modern immunotherapy agents.

#### 2.7.1. Cemiplimab

Cemiplimab is a monoclonal antibody that targets PD-1. It was the first anti-PD-1 drug to be FDA approved for frontline management of patients with locally advanced or metastatic CSCC [64]. Cemiplimab was evaluated in two open-label, multicenter studies: Study 1423 had 26 enrolled patients [14,65], and Study 1540 (EMPOWER-CSCC 1) had 193 enrolled patients [14,66,67,68]. In the phase I study, durable responses were observed in 50% of the 26 patients treated. These results were confirmed in the EMPOWER-CSCC 1 trial, in which an ORR of 50% was achieved; 7% of the study cohort achieved a complete response (CR). Key exclusion criteria included SOTR; ongoing or recent immunosuppressant use for autoimmune disease within the last 5 years; patients previously treated with ICI; history of hepatitis/HIV infection; or Eastern Cooperative Oncology Group performance status ≥ 2. Cemiplimab was noted to have an acceptable safety profile with a discontinuation rate of only 7% and a similar rate of adverse events to other anti-PD-1 drugs [14]. Finally, cemiplimab was evaluated in a 2018 study to obtain real-world data on its efficacy and safety. In this retrospective multicenter study of 131 patients, the ORR was 58% with a discontinuation rate of 9.2%—comparable to previous clinical trial data [69].

#### 2.7.2. Pembrolizumab

Pembrolizumab is a monoclonal antibody that targets PD-1. It is approved for frontline management of patients with recurrent, metastatic, or locally advanced HNCSCC that cannot be cured with surgery or RT [16]. Pembrolizumab efficacy was evaluated in 2 open-label, single-arm, phase II multicenter studies: KEYNOTE-629 enrolled 105 patients [16] and CARSKIN enrolled 39 patients in the initial cohort along with 57 patients in the expansion cohort [70]. In KEYNOTE-629, most of the patients received prior systemic therapy (87%). The ORR was 34%, CR rate was 4%, and partial response (PR) rate was 31%. Pembrolizumab was well tolerated, and the observed toxicities were similar to other pembrolizumab clinical trials [16].

In the investigator-initiated CARSKIN trial, treatment-naïve patients with advanced CSCC were enrolled and treated for up to 2 years. The ORR was 42%, CR was 7%, and PR was 35%. Additionally, the ORR was significantly greater (55%) among patients with PD ligand-1 (PD-L1) positivity, compared to patients with PD-L1 negative tumors (17%). In the primary cohort patients who were treated up to week 15, the ORR was 41%. None of the patients recurred during the follow-up observation period of 22.4 months. One developed a new primary HNCSCC. Finally, the median PFS and OS were 29 weeks (confidence interval (CI), 15 weeks–NR) and 108 weeks (CI, 61 weeks–NR), respectively [70]. Interestingly, some patients had a response up to 72 weeks after starting treatment.

### 2.8. Predictors of Treatment Response to Immunotherapy in HNCSCC

Presently there are no predictive ICI biomarkers to assess whether patients are responding well to treatment or to guide decision making for patients with HNCSCC [9]. Response to cemiplimab is not dependent on PD-L1 status, and durable responses have been observed in both low and high PD-L1 expression groups [15]. A similar finding was observed with pembrolizumab in KEYNOTE-629. There was no significant relationship observed between PD-L1 expression and treatment response, although there was a tendency toward improved ORR in subgroups with a PD-L1 positive score of 1 or more in the combined analysis of patients with metastatic and locally advanced HNCSCC [71]. Given these findings, PD-L1 status should not influence treatment decisions regarding systemic immunotherapy.

The role of TMB as a predictive biomarker for HNCSCC patients remains unclear. A study published by Goodman et al. involving SCC patients concluded that 2 characteristics, cutaneous origin and higher TMB, correlated with improved outcomes for PD-L1 blockade [72]. However, this study had several limitations including a small sample size. Additionally, further study analysis demonstrated a lack of statistical significance between TMB and clinical benefit of immunotherapy, limiting our interpretation of such findings [9].

### 2.9. Special Considerations

Not only are immunocompromised patients at increased risk for HNCSCC, but immune status has been demonstrated to negatively affect disease outcomes. A multicenter study by Manyam et al. showed significantly lower locoregional recurrence-free survival (47.3% vs. 86.1%) and PFS (38.7% vs. 71.6%) in immunosuppressed compared to immunocompetent patients [73]. A retrospective study by Tam et al. demonstrated similar findings, as immunocompromised status was associated with lower disease-free survival [74].

Additionally, in the SOTR patient population, the type of immunosuppressant used may influence the development of CSCC. Calcineurin inhibitors are known to increase the likelihood of CSCC development [75,76]. In order to mitigate this risk, there has been a trend toward the use of mammalian target of rapamycin (mTOR) inhibitors in SOTR. Indeed, the use of mTOR inhibitors was noted to increase disease-free survival in kidney transplant patients with a history of CSCC [77].

Finally, immunotherapy clinical trials have historically excluded SOTR patients, due to the possibility of graft rejection following ICI-induced immune activation. Therefore, a risk-benefit discussion should take place with SOTR patients before starting immunotherapy because of the significant potential for allograft rejection, resulting in the need for dialysis and/or death [9]. Currently, the safety and effectiveness of immunotherapy in SOTR is being investigated in a clinical trial involving kidney transplant recipients who have been diagnosed with advanced cutaneous malignancies (NCT03816332).

## 3. Immunotherapy Future Directions

### 3.1. Neoadjuvant Setting

Currently, there are no approved ICI drugs for treatment of HNCSCC in the neoadjuvant or adjuvant setting. Clinical trials are ongoing, investigating the use of ICIs (both alone and in combination) prior to surgery. However, risks associated with this approach include a delay in surgical treatment, which may result in an unresectable tumor and increase the risk of adverse events. In a study by Ferraroto et al. involving 20 patients with recurrent and resectable stage II-IV HNCSCC, 2 cycles of cemiplimab prior to surgery resulted in 11 pathologic CRs and 3 major pathologic responses [78]. A follow-up study published in 2022, involving 79 patients with operable stage II-IV HNCSCC, found that up to 4 doses of cemiplimab prior to surgery was associated with 51% of patients achieving pathologic CRs [79]. Interestingly, 68% of patients with a clinical partial response were found to have a complete pathological response, clinically underestimating the activity of cemiplimab in this patient population.

### 3.2. Adjuvant Setting

Previous studies in metastatic melanoma patients demonstrated that lower disease burden was associated with an increased likelihood of survival following treatment with PD-1 inhibition [80,81]. Based on these findings, it has been hypothesized that treatment of residual microscopic HNCSCC disease following surgery with PD-1 blockade may lead to improved recurrence-free and overall survival. Currently, 2 phase III studies (NCT03969004 and NCT03833167) are investigating the effectiveness of cemiplimab and pembrolizumab, respectively, in patients with high-risk or locally advanced HNCSCC who have been treated with surgery followed by RT. Given the lack of published study data, the use of immunotherapy in the adjuvant setting remains limited to patients enrolled in clinical trials [9].

## 4. New Immunotherapy Drugs and Combinations

Novel treatment approaches are needed to improve outcomes in patients with HNCSCC and to overcome drug resistance [6]. These approaches include the use of new ICIs, combination therapies, and oncolytic viruses.

### 4.1. Nivolumab

While not officially approved for use in patients with non-melanoma skin cancer, other ICIs may be effective in treating patients with advanced CSCC. Their efficacy thus far has been limited to published case reports and case series.

Nivolumab, a monoclonal antibody targeting PD-1, was assessed in 3 patients with recurrent CSCC who had previously been treated with chemotherapy. A PR was noted in 2 patients, and 1 patient experienced stable disease following 3 months of treatment [82]. Chen et al. described a patient with poorly differentiated advanced CSCC who achieved CR following treatment with nivolumab and cetuximab [83]. Presently, there are several ongoing clinical trials (NCT03834233, NCT04204837) evaluating the efficacy of nivolumab in the treatment of locally advanced and metastatic CSCC [84].

### 4.2. Ipilimumab

Ipilimumab is a CTLA-4 inhibitor approved for use in metastatic melanoma patients. Similar to nivolumab, its efficacy has been limited to published case reports. Day et al. described a patient with metastatic CSCC, previously treated with chemotherapy, who achieved a CR following 4 cycles of ipilimumab [85]. We have personally treated 3 such refractory patients and observed a major response to treatment in each case (unpublished data).

### 4.3. Immunotherapy and Radiation Therapy

Another innovative strategy is the combination of immunotherapy with RT. Radiation-induced damage to cells has several effects on the immune system, including T-cell priming and upregulation of immune checkpoint molecules on tumor cells, which in turn can facilitate increased immune-mediated cell death when combined with ICIs [86]. This treatment approach is currently being evaluated in the UNSCARRed study, investigating the effects of RT and avelumab in patients with unresectable CSCC (NCT03737721).

### 4.4. Immunotherapy and EGFR Inhibitors

The I-TACKLE trial—an open-label, nonrandomized, phase II trial in 43 patients with locally advanced or metastatic CSCC conducted in 3 Italian centers—investigated the addition of an anti-EGFR agent cetuximab to pembrolizumab to counteract pembrolizumab resistance. Forty-four percent of patients experienced a response to pembrolizumab with a cumulative ORR of 63%. Twenty-one patients had primary resistance to pembrolizumab and received the combination therapy [87]. The addition of cetuximab led to a response rate of 38% and is hypothesized to produce a synergistic effect as it contributes to NK cell activation, which in turn activates negative feedback controls via increased expression of PD-1, PD-L1, and CTLA-4 [88].

### 4.5. Oncolytic Viruses

**Figure 2:**
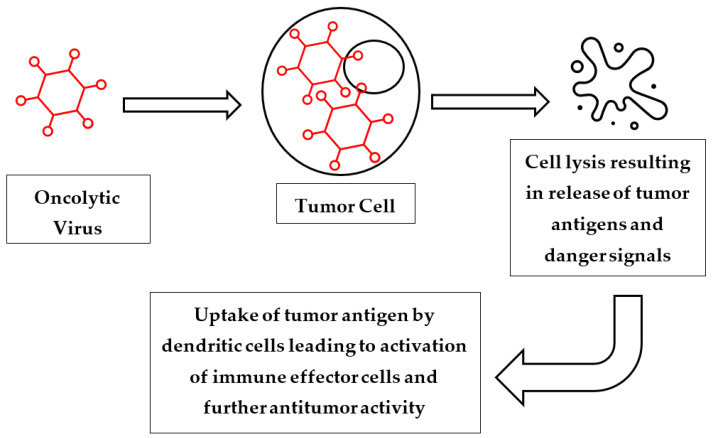
Mechanism of action of oncolytic viruses. Injection of oncolytic virus into tumor cell. This results in viral replication, and in turn leads to cell lysis and death. Release of tumor cell contents stimulates activation of immune effector cells via antigen presentation by dendritic cells, leading to both local and systemic antitumor effects.

Oncolytic viruses (OVs) are injected into tumors and exert both local and systemic antineoplastic effects within the body. Locally, OVs selectively infect and grow within the injected tumor, resulting in tumor lysis and cell death [89]. Tumor lysis leads to the release of antigens and danger signals, which promote enhanced dendritic cell antigen presentation, T-cell priming, and T-cell mediated cytotoxicity within the injected tumor. Additionally, OVs can promote the migration of tumor-specific T cells to uninjected tumor cells and exert distant antineoplastic effects [90]. Finally, increased interferon (IFN) gamma signaling leads to upregulation of PD-1 on host T cells and PD-L1 on tumor cells, resulting in an augmented antineoplastic effect when combined with PD-1 blockade [91] (Figure 2).

RP1 is a genetically modified herpes simplex OV. Niu et al. reported promising results in the phase I/II IGNYTE trial, in which patients with HNCSCC and other tumor types achieved a CR rate close to 50% when treated with RP1 monotherapy or in combination with nivolumab. Tumor regression was observed in both injected and non-injected distant lesions following RP1, suggestive of a systemic treatment response [92]. A phase II trial investigating the use of RP1 alone and with cemiplimab in patients with advanced CSCC (NCT04050436) is ongoing. Finally, RP1 is being investigated in a phase IB/II trial of SOTR with CSCC and other advanced cutaneous malignancies (NCT04349436).

Talimogene laherparepvec (T-VEC) is another genetically modified herpes simplex OV currently being studied in CSCC. A recent phase II study interim analysis reported 100% ORR in 7 patients with low-risk CSCC treated with T-VEC [93]. Ongoing clinical trials are investigating combination therapy with T-VEC, including the use of nivolumab (NCT03714828) and panitumumab (NCT04163952).

### 4.6. Other Therapies

Two studies are investigating the use of photoimmunotherapy in the treatment of patients with CSCC. This involves the injection of an antibody-dye conjugate, followed by activation with a particular wavelength of light. RM-1995 is composed of an antibody that targets CD25, a receptor with increased expression by regulatory T cells (Treg). This drug conjugate is activated when illuminated by red light, and targets Treg cells within tumors, thus augmenting the antitumor immune response. RM-1995 is given as a single agent as well as in combination with pembrolizumab (NCT05220748). In the other study, ASP-1929 is composed of an EGFR antibody that is also activated by the illumination of red light. ASP-1929 will be given in combination with cemiplimab (NCT04305795).

Another study is exploring the use of an intra-tumoral vaccine injection expressing a DNA plasmid known as IFX-Hu2.0 (NCT04160065). This results in expression of a streptococcal membrane protein within the lesion, which in turn stimulates the immune system and produces a favorable environment for ICI therapy. This phase I study plans to enroll 20 patients with advanced non-melanoma skin cancers (accrual is ongoing).

Additionally, B7-H3 has emerged as a promising molecular target for immunotherapy-based studies. It is an immune checkpoint protein, which exerts inhibitory effects on the immune system via suppression of T-cell activation and proliferation. It is highly expressed in various solid tumors, yet has limited expression in normal tissues. Increased B7-H3 expression has been shown to promote immune escape and tumor growth in head and neck squamous cell carcinoma (HNSCC) stem cells [94]. Furthermore, a 2018 study found B7-H3 to be highly expressed, particularly in immunocompetent patients with CSCC [95]. While there are no current clinical trials involving CSCC patients, B7-H3 has been found to be an effective target in other advanced cancers including HNSCC and melanoma. A recently published phase I/II study found the combination of enoblituzumab, a B7-H3 monoclonal antibody, and pembrolizumab was highly effective (ORR 33%) and well tolerated in patients with advanced solid tumors [96]. Furthermore, interim results from a phase I study of enoblituzumab in combination with ipilimumab in refractory cancers suggested it was well tolerated; however, final study results have not been published yet (NCT02381314).

Finally, several studies are exploring the use of cytokines in combination with immunotherapy, including one phase IB/IIA study evaluating the use of interleukin-7 (IL-7) in combination with atezolizumab in patients with advanced cutaneous malignancies (NCT03901573). IL-7 is critical for the development of naïve and memory T cells, which in turn facilitate an antitumor immune response [6]. 

## 5. Summary and Conclusions

CSCC is common, and most cases are diagnosed at an early stage and associated with a favorable prognosis. Advanced-stage presentations are rare but require multidisciplinary care due to the complexity of the disease, the patients’ comorbidities, and the need for systemic treatment in an often-frail older population. Immunotherapy has transformed the care of advanced or mHNCSCC patients due its favorable side effect profile, durable treatment response, and improved OS rates. Anti-PD-1 drugs remain the backbone of clinical investigation, and new combinations are being tested, including the co-administration of RT, EGFR antagonists, or OV. Additionally, the activity, efficacy, and safety of anti-PD-1 drugs are currently being evaluated in the neoadjuvant and adjuvant settings for patients with advanced CSCC (Table 1 and Table 2).

## Figures and Tables

**Table 1:** Current Immunotherapy Clinical Trials in HNCSCC.

Interventions	Sponsor	Study Design	Conditions	Status	NCT
**Neoadjuvant** **Pembrolizumab**	**Queensland Health**	**Phase II**	**●HNCSCC**	**Active,** **not recruiting**	**NCT05025813**
**Pembrolizumab &** ** Quad Shot RT**	**Wake Forest**	**Phase II**	**●HNCSCC**	**Active,** ** recruiting**	**NCT04454489**
**Pembrolizumab,** ** BAY 1895344, & RT**	**National Cancer** **Institute**	**Phase I**	**●HNCSCC** **●Oral cavity SCC** **●Oropharyngeal SCC** **●Hypopharyngeal SCC** **●Laryngeal SCC**	**Active,** ** recruiting**	**NCT04576091**
**Pembrolizumab, CIMAvax vaccine, & Nivolumab**	**Roswell Park Cancer Institute**	**Phase I/II**	**●HNCSCC** **●Advanced NSCLC** **●HNSCC**	**Active,** ** recruiting**	**NCT02955290**
**Pembrolizumab &** **Personalized Neoantigen Peptide-Based Vaccine**	**Mayo Clinic**	**Phase I**	**●Advanced** **Solid Tumors**	**Active,** ** recruiting**	**NCT05269381**
**Pembrolizumab & Sonidegib**	**Mayo Clinic**	**Phase I**	**●Advanced** **Solid Tumors**	**Active,** ** recruiting**	**NCT04007744**
**ONCR-177 (oncolytic virus) Alone & Combined with Pembrolizumab**	**Oncorus**	**Phase I**	**●Advanced** **Solid Tumors** **●Liver Metastases of Solid Tumors**	**Active,** ** recruiting**	**NCT04348916**
**TransCon TLR7/8 Agonist Alone & Combined with Pembrolizumab**	**Ascendis Pharma**	**Phase I**	**●Advanced** **Solid Tumors** **●HNSCC** **●CSCC**	**Active,** ** recruiting**	**NCT04799054**
**Neoadjuvant** **Atezolizumab**	**UC Davis**	**Phase II**	**●HNCSCC**	**Active,** ** recruiting**	**NCT05110781**
**Beta IL-2**	**Medicenna Therapeutics**	**Phase I/II**	**●Advanced** **Solid Tumors**	**Active,** ** recruiting**	**NCT05086692**

Abbreviations: RT: radiotherapy; HNCSCC: head and neck cutaneous squamous cell carcinoma; SCC: squamous cell carcinoma; NSCLC: non-small cell lung cancer; HNSCC: head and neck squamous cell carcinoma; TLR: Toll-like receptor; IL-2: interleukin-2.

**Table 2:** Immunotherapy Agents with Activity in CSCC.

Author	Agents	Study Type	# of Patients	Median Follow-Up (Months)	ORR
**Rischin et al. [66,67,68]**	**Cemiplimab**	**Prospective**	**193**	**15.7**	**46.1%**
**Ferrarotto et al. [78]**	**Cemiplimab**	**Prospective**	**20**	**22.6**	**30%**
**Gross et al. [79]**	**Cemiplimab**	**Prospective**	**79**	**NR**	**68%**
**Grob et al. [16]**	**Pembrolizumab**	**Prospective**	**105**	**11.4**	**34%**
**Maubec et al. [70]**	**Pembrolizumab**	**Prospective**	**57**	**22.4**	**42%**
**Bossi et al. [87]**	**Pembrolizumab+cetuximab**	**Prospective**	**43**	**24**	**63%**
**Niuet et al. [92]**	**RP1 (OV)+nivolumab**	**Prospective**	**15**	**NR**	**60%**
**Curiel et al. [93]**	**T-VEC (OV)**	**Prospective**	**7**	**NR**	**100%**

Abbreviations: ORR: overall response rate; NR: not reported; OV: oncolytic virus; T-VEC: talimogene laherparepvec.
